# Personality Traits in Miners with Past Occupational Elemental Mercury Exposure

**DOI:** 10.1289/ehp.7863

**Published:** 2006-01-18

**Authors:** Darja Kobal Grum, Alfred B. Kobal, Niko Arnerič, Milena Horvat, Bernard Ženko, Sašo Džeroski, Joško Osredkar

**Affiliations:** 1 Department of Psychology, Faculty of Arts, University of Ljubljana, Slovenia; 2 Department of Occupational Medicine, Idrija Mercury Mine, Idrija, Slovenia; 3 Clinical Institute of Occupational, Traffic and Sports Medicine, University Medical Centre, Ljubljana, Slovenia; 4 Departments of Environmental Sciences and; 5 Knowledge Technologies, Jozef Stefan Institute, Ljubljana, Slovenia; 6 University Medical Centre Ljubljana, Clinical Institute of Clinical Chemistry and Biochemistry, Ljubljana, Slovenia

**Keywords:** depression, elemental mercury, ex-mercury miners, Hg, negative self-concept, occupational exposure, personality traits

## Abstract

In this study, we evaluated the impact of long-term occupational exposure to elemental mercury vapor (Hg^0^) on the personality traits of ex-mercury miners. Study groups included 53 ex-miners previously exposed to Hg^0^ and 53 age-matched controls. Miners and controls completed the self-reporting Eysenck Personality Questionnaire and the Emotional States Questionnaire. The relationship between the indices of past occupational exposure and the observed personality traits was evaluated using Pearson’s correlation coefficient and on a subgroup level by machine learning methods (regression trees). The ex-mercury miners were intermittently exposed to Hg^0^ for a period of 7–31 years. The means of exposure-cycle urine mercury (U-Hg) concentrations ranged from 20 to 120 μg/L. The results obtained indicate that ex-miners tend to be more introverted and sincere, more depressive, more rigid in expressing their emotions and are likely to have more negative self-concepts than controls, but no correlations were found with the indices of past occupational exposure. Despite certain limitations, results obtained by the regression tree suggest that higher alcohol consumption per se and long-term intermittent, moderate exposure to Hg^0^ (exposure cycle mean U-Hg concentrations > 38.7 < 53.5 μg/L) in interaction with alcohol remain a plausible explanation for the depression associated with negative self-concept found in subgroups of ex-mercury miners. This could be one of the reason for the higher risk of suicide among miners of the Idrija Mercury Mine in the last 45 years.

The central nervous system is the critical organ for elemental mercury vapor (Hg^0^) exposure [World Health Organization (WHO) 1976]. Postmortem studies (Byrne et al. 1995; Falnoga et al. 2000; Kosta et al. 1975) have shown that the accumulation of Hg in the brains of ex-mercury miners was very high even several years after exposure. Strong Hg accumulation and retention were found particularly in the hippocampus, cerebellar cortex, nucleus dentatus, pituitary, and the pineal gland.

Long-term occupational exposure to Hg^0^ is associated with symptoms of erethism, characterized by irritability, depression, introversion, apprehension, loss of self-confidence, and other nonspecific symptoms (WHO 1976, 1991, 2003). These symptoms also characterized the clinical pictures of Hg^0^-intoxicated miners of the Idrija Mercury Mine (Hribernik 1950; Kobal 1975, 1991).

Only a few studies have evaluated the residual, mostly neurologic, neurophysiologic, and neuropsychologic effects associated with remote occupational Hg^0^ exposure (Albers et al. 1988; Andersen et al. 1993; Ellingsen et al. 1993; Letz et al. 2000; Mathiesen et al. 1999). To our knowledge, only the study of Letz et al. (2000) evaluated residual mood effects in workers previously exposed to Hg^0^, but the authors state that no changes were observed.

An epidemiologic study (Boffetta et al. 1998) of the causes of death among miners in certain mercury mines revealed an increased mortality rate due to suicide among the miners of the Idrija Mercury Mine. During the long-term medical monitoring of miners exposed to Hg^0^ (Hribernik 1950; Kobal 1975, 1991), changes to miners’ personalities were observed several times. Our purpose in the present study is to evaluate the impact of long-term occupational exposure to Hg^0^ on the personality traits reported by ex-miners in the Eysenck Personality Questionnaire (EPQ) and the Emotional States Questionnaire (ESQ) in the period after exposure. We focused particularly on the relationship between past occupational Hg^0^ exposure and appearance of depressive mood and negative self-concept among ex-miners, which could increase the risk of suicide.

## Materials and Methods

### Subjects.

One hundred twenty males examined in the study were divided into ex-mercury miners and control groups. The ex-mercury miners were recruited from the Idrija Mercury Mine. The inclusion criteria for ex-mercury miners were age younger than 64 years, exposure to Hg^0^ for at least 3 years or 12 exposure cycles, and no exposure to Hg^0^ in at least the past 12 or 8 months but urinary mercury (U-Hg) excretion below 15 μg/L at last exposure. Exposure cycle is defined as number of days per month of work in Hg^0^-contaminated work areas in the mine. These criteria were met by 60 ex-mercury miners who were identified from the company’s medical records and biological monitoring data. The control workers—60 males—were taken from “mercury-free” forestry occupations. These workers performed jobs as choppers and transport workers and were located in neighboring municipalities to match the ex-mercury miners as closely as possible for socioeconomic factors. The workload in their jobs was similar to those of ex-mercury miners, which varied from 5.0 to 9.0 Kcal/min (Kobal 1991). Ex-miners were additionally exposed to silica dust and periodically to radon and its daughters (the most exposed group of miners did not receive more than 8 mSv for 2,000 hr of work a year). The subjects of both groups worked only in day shifts.

The final selection of the study population was based on medical examinations and some biologic analyses conducted at the time of the survey. The following criteria were applied: ex-mercury miners and control workers were neither currently nor previously exposed to lead, cadmium, or solvents, and the medical history and medical examinations of the control workers and ex-mercury miners did not reveal any neurologic or psychiatric affections (e.g., alcoholism, head trauma, meningitis, epilepsy, episodes of severe depression), metabolic disorders, hepatic or renal disease, or medical treatment possibly influencing the results of psychologic tests (e.g., β-blocker, antidepressive agent, etc.).

Fourteen subjects did not meet the above selection criteria and were excluded from the definitive population, which at the end consisted of 53 ex-mercury miners and 53 control group workers. Two ex-miners were excluded because of hypertension and renal disease, two because of alcohol abuse, one because of episodes of cerebral ischemia, and one because of metabolic disorders. Two control workers were excluded because of sick leave, two because of unwillingness to participate, one because of metabolic disorders, and one because of alcohol abuse. One ex-miner and one control worker were excluded from psychologic examinations because of their low general intellectual level, which caused obvious difficulties in understanding instructions during the test session.

The study group of ex-mercury miners was examined after exposure to Hg^0^. This group consisted of 20 retired miners and 33 still active but Hg^0^-unexposed miners (mining and production of Hg was stopped in 1994). In the past, mercury miners were intermittently exposed during working cycles to native Hg at air Hg^0^ concentrations varying from 0.05 to > 1.00 mg/m^3^. When Hg^0^ exposure exceeded the occupational exposure limit of 0.1 mg/m^3^, miners used personal protective equipment, for example, half masks or Racal helmets with Hg^0^-absorbing filters (Kobal and Dizdarevič 1997).

The study was conducted with the approval of the State Ethical Commission and in accordance with the ethical standards laid down in the Helsinki Declaration. All participants gave their written informed consent before being included in the study.

### Medical and psychologic examination.

To standardize as much as possible with respect to work shift schedules, we conducted the examinations once a week on Friday. The medical examination included determination of general clinical status of examinees’ medical histories and lifestyle habits (smoking, alcohol consumption). Examinations were performed by a physician–occupational medicine specialist with long-term experience in the health surveillance of workers exposed to Hg^0^, in line with the standard clinical methodology. The overall examination time was approximately 120 min per subject, including the time required for giving instructions on how to perform the tests.

The self-reported mean alcohol consumption was converted to units of pure alcohol in milliliters per day (Kobal et al. 2004). A dental amalgam score was calculated using the methodology proposed by Aposhian et al. (1992). The examination included venous blood and urine sampling (8-hr urine samples collected from 2100 to 0600 hr) for determination of *a*) blood total Hg (B-THg) and U-Hg; *b*) selected hematologic data (erythrocytes, erythrocyte sedimentation rate, hematocrit, hemoglobin in blood, leucocytes, mean corpuscular hemoglobin, reticulocytes, thrombocytes, differential leukocyte count); *c*) selected blood and urinary data on the kidney and urinary tract (creatinine and urea in blood, urine test strip analyses, urine albumin and creatinine); and *d*) serum γ-glutamyltransferase (GGT), aminotransferases, bilirubin, blood glucose, and c-reactive protein. All participants completed a Slovene translation (Lojk 1981) of the EPQ (Eysenck and Eysenck 1975) and the ESQ (Lamovec 1989). The collected data proved the EPQ questions to be appropriate for the Slovene population, as they were for the British and Danish (Eysenck and Eysenck 1975; Mortensen et al. 1996) populations. The personality structure appears to be similar in spite of the differences in nationality (Lojk 1981). The self-administrated Emotional State scales include 54 emotional descriptors rated on a 4-point scale from “none at all” to “extreme.” The items consist of five emotional states: depression, contentment, aggression, indifference (tendency to emotional rigidity), and self-concept (positive and negative). The ESQ has a very similar theoretical view, as presented in the study by Sjöberg and Swensson (1976) and based on the study results of the Slovene population (Lamovec 1988). The factor analysis of primary emotions showed that the depression indicated in our questionnaire conforms with the clinical description of this condition, which, apart from depression, also includes elements of anxiety (Lamovec 1989). The estimates of the degree of reliability could be arbitrary to some extent, but some of the values of Cronbach α could hardly be rated as relatively high. However, the total measures of the Cronbach α-coefficient (Vogt 1993) and the Guttman split-half coefficient are beyond the lower level of acceptable reliability (Table 1). It could be suggested that, because reliability is associated with accuracy of the test, ESQ may be ranged among those psychometric tests with fairly good reliability.

### Assessment of exposure.

Environmental and biological data on the group of miners studied have been collected from 1959 onward from daily reports on Hg^0^ measurements in the work-place, personal medical records, and biological monitoring data. On the basis of their exposure records, the following environmental indices of Hg^0^ occupational exposure were calculated for each miner: *a*) years of work in the mercury mine (years of exposure); *b*) cycles of exposure (intervals of work exposed to Hg^0^); and *c*) average time-weighted (ATW) air Hg^0^ concentration expressed in milligrams Hg^0^ per cubic meter of air (Kobal and Dizdarevič 1997). Hg^0^ in the air within the mine was generally determined by ultraviolet photometry using two portable instruments (model K-23 mercury vapor meter, Beckman Coulter, Fullerton, CA, USA; mercury indicator, Beckman Coulter UK Ltd., Shawcity, UK) designed to detect minute concentrations as described elsewhere (Kobal and Dizdarevicč 1997).

The miners were biologically monitored by U-Hg analyses. High variations of U-Hg excretion are characteristic for the intermittent type of exposure observed in miners of the Idrija Mercury Mine (Kobal 1991). To avoid overestimation of the integral internal doses received during miners’ occupational exposures, we considered not only the peak U-Hg levels of the cycles but also all U-Hg measurements obtained during and after the exposure cycles. The results of more than 5,400 U-Hg measurements (spot urine samples) were identified from the 53 ex-mercury miners. On the basis of these data, the following individual biological indices of occupational exposure were calculated: *a*) geometric mean of cycle U-Hg level, calculated from all urine samples determined during and after the cycles of exposure expressed in micrograms Hg per liter; *b*) the geometric mean of cycle peak U-Hg level, calculated from all cycle peak-Hg levels expressed in micrograms Hg per liter; *c*) the cumulative U-Hg level (the sum of U-Hg levels of all cycles) expressed in micrograms Hg per liter; *d*) cumulative peak U-Hg level (the sum all cycle peak U-Hg levels); and *e*) the U-Hg level at last exposure expressed in micrograms Hg per liter. The actual background exposure to inorganic Hg was evaluated by determining present and U-Hg. The potential methyl mercury exposure (from fish intake) was evaluated by determining blood methylmercury.

### Biological analyses.

B-THg and U-Hg were determined by cold-vapor atomic absorption spectrophotometry (CVAAS). The limit of detection of B-THg was 0.05 ng Hg/mL of blood. The actual U-Hg concentration was analyzed in an 8-hr urine sample collected in a metal-free polypropylene bottle during the night (2200–0600 hr). The detection limit of Hg in a 0.5-mL urine sample was 0.05 ng (Horvat et al. 1986, 1991). Before 1970, U-Hg was analyzed using the dithizone method, and afterward using the above-mentioned CVAAS technique expressed in micrograms per liter. Creatinine in urine was measured on a Roche/Hitachi 917 automated biochemical analyzer (Roche, Mannheim, Germany). The GGT, asparate aminotransferase (AST), and alanine amino-transferase (ALT) as alcohol abuse markers (Joachim 1995) and other basic routine biochemical and hematologic parameters were determined by applying the usual clinical biochemical methods (data not shown).

### Data analyses.

The group differences in all observed parameters were evaluated by applying a one-way analysis of variance using ANOVA software. The relationship between exposure and other variables was evaluated by Pearson’s correlation coefficient, which reflects the degree of linear relation between two sets of data. The SPSS for Windows (version 11.0.1; SPSS Inc., Chicago, IL, USA) software package was used for all computations. Machine learning methods were used to find possible explanations of associations between the target variables (personality traits) and biological indicators of occupational Hg^0^ exposure in combination with covariables on the subgroup level. We used these methods because they produce interpretable models, as opposed to most other nonlinear modeling procedures, which give us models that are hard to interpret (so-called black box models). Machine learning methods used are model trees (Quinlan 1992), which are a generalization of regression trees (Breiman et al. 1984).

Regression trees are a formalism for representation of piecewise constant functions, whereas model trees are more general and are a formalism for representation of piecewise linear functions. Like classic regression equations, model trees predict the value of a dependent variable (called target variable or class) from the values of a set of independent variables (called attributes). Data represented in the form of a table can be used for learning or construction of a model tree. In the table, each row (example or subject) has the form (*x*_1_, *x*_2_, . . . , *x_n_*, *y*), where *x_i_* are values of *n* attributes (e.g., subjects’ ages, daily consumption of alcohol, etc.) and *y* is the value of the target variable (e.g., the ESQ depression score). Unlike classic regression approaches, which find a single equation for a given set of data, model trees partition the space of examples into axis-parallel rectangles and fit a model to each of these partitions. A model tree has a test in each inner node that tests the value of a certain attribute, and in each leaf a model for predicting the target variable: the model can be a linear equation or merely a constant. Given a new example (subject) for which the value of the target variable should be predicted, the tree is interpreted from the root. In each inner node, the prescribed test is performed, and according to the result, the corresponding left or right subtree is selected. When the selected node is a leaf, the value of the target variable for the new example is predicted according to the model in the leaf.

A number of systems are available for inducing regression and model trees, such as RETIS (Karalič 1992) and M5 (Quinlan 1992). The latter is one of the best-known systems for regression and model tree induction. We used the M5′ system (Wang and Witten 1997), a reimplementation of M5 within the WEKA software package (Witten and Frank 1999). The parameters of M5′ were set to their default values.

A model tree for each of the selected target variables of personality traits (only target variables with group differences of *p* < 0.01 have been considered) was induced on the following features (independent variable): groups (ex-mercury miners; underground work, controls; work in the open), subgroups of ex-mercury miners (active but not exposed to Hg^0^ miners; retired miners), age, residence (municipality of Idrija, other location, town center, hillside), dental amalgam score, cigarettes per day, alcohol consumption (milliliters per day), years of work in the mercury mine (years of exposure), work cycles of Hg exposure (number), the geometric mean of cycle U-Hg level in micograms per liter, the geometric mean of peak cycle U-Hg level in micograms per liter, cumulative U-Hg and cumulative peak U-Hg levels in micograms per liter, U-Hg level at last exposure micograms per liter, and time since last exposure in days (exposure-free interval). Those miners with an exposure-free interval of < 12 months were excluded from evaluation. Model trees for depression score and negative self-concept score were induced on subjects from both groups (miners and controls), whereas for modeling the lie score, only the miners were considered. Only those models that, in addition to indicators of occupational exposure, showed characteristics common to both groups were used. To stress the significance of last exposure in these evaluations, all U-Hg values of last exposure < 10 μg/L were ignored (five miners). Each model was evaluated using Pearson’s correlation coefficient on the training data only because we were interested in possible explanations of group differences that were already found to be significant.

## Results

### Characteristics of ex-mercury miners and controls.

Despite their similar socioeconomic conditions, lifestyle, and same biologic characteristics, the groups differed with respect to the location of their work, which is performed underground by miners and above ground by controls, as well as by their occupational exposure to Hg^0^ and some other previously mentioned pollutant in the mine.

The observed groups did not differ in mean age, body mass index (BMI), dental amalgam score, fish intake, cigarette and alcohol consumption (Table 2). The mean consumption of alcohol tended to be higher in miners (35 vs. 22 mL/day). The number of alcohol consumers with > 20 mL/day was higher in miners (28% vs. 19%), but no significant differences between the two groups were detected (*p* > 0.05). At these levels of alcohol consumption, induction of the microsomal ethanol-oxidizing system and increased activity of certain liver enzymes may be expected (Marks et al. 1996), but on a group level no differences in the mean serum GGT and AST levels (*p* > 0.05) were detected between miners and controls (data not shown). A slight correlation between alcohol consumption and serum GGT (*r* = 0.31, *p* = 0.034) and AST (*r* = 0.48, *p* = 0.000) was found in miners.

The groups did not differ in mean B-THg concentrations, which represents the actual exposure to Hg^0^ and methylmercury (fish intake). The dental Hg amalgam score in controls correlated with B-THg (*r* = 0.30, *p* = 0.04); no such correlation was found in the group of ex-mercury miners. The U-Hg concentration (micrograms per gram creatinine) was significantly higher (*p* = 0.003) in the miner group than in the control group because of the higher U-Hg excretion in ex-mercury miners whose interval since last exposure was < 6 years.

### Occupational Hg^0^ exposure status.

Mercury miners were observed in the period after long-term intermittent exposure to Hg^0^, which lasted 7–31 years. Before the present observations, the miners had no longer been exposed to Hg^0^ for on average 5.9 years (range, 8–336 months). Because of job rotations (from Hg^0^-contaminated to noncontaminated work-places), the miners were periodically, in cycles, exposed to Hg^0^ several times a year (Kobal and Dizdarevič 1997). The total number of exposure intervals—cycles of exposure—varied from 13 to 119. On average, the miners’ cycles of Hg^0^ exposure lasted 19 days (range, 3–34 days). The biological indices of occupational exposure presented in Table 3 were relatively high despite the use of personal protective equipment. The geometric mean of cycles U-Hg levels varied from 20 to 120 μg/L, yet intraindividual variability was very high, with a coefficient of variability in the range of 53–104%.

The best positive correlation with the biological indices of exposure was shown by the cycles number of exposure. Its correlation with the cycle U-Hg level (*r* = 0.395, *p* = 0.003), the cycle peak U-Hg level (*r* = 0.371, *p* = 0.006), and the U-Hg level at last exposure (*r* = 0.374, *p* = 0.005) was moderate but significant. A relatively good correlation was found between U-Hg at last exposure and the mean cycle U-Hg concentration (*r* = 0.448, *p* = 0.001). No positive correlation was found between the present U-Hg level and past external or biologic occupational exposure indices.

### Psychologic evaluation.

Table 4 presents the EPQ. A comparison of the group of ex-mercury miners and the control group revealed a lower mean score of extraversion in the miners group (*p* = 0.017). The average score on the lie scale was also lower in the group of miners (*p* = 0.003).

Table 5 presents the ESQ. The average scores for depression and negative self-concept were significantly higher (*p* < 0.01) in the ex-mercury miners’ group than in the controls. The indifference average score also tended to be higher in miners (*p* = 0.025) than in controls. We found no correlation of lie, depression, negative self-concept score, or other EPQ and ESQ variable scores with past external or biological occupational exposure indices evaluated using Pearson’s correlation coefficient. A positive significant correlation between the depression score and the negative self-concept score was found in miners (*r* = 0.757, *p* = 0.009) and controls (*r* = 0.807, *p* = 0.000). No correlation was found between alcohol consumption and lie score, or between depression score and negative self-concept score.

**Figure 1:**
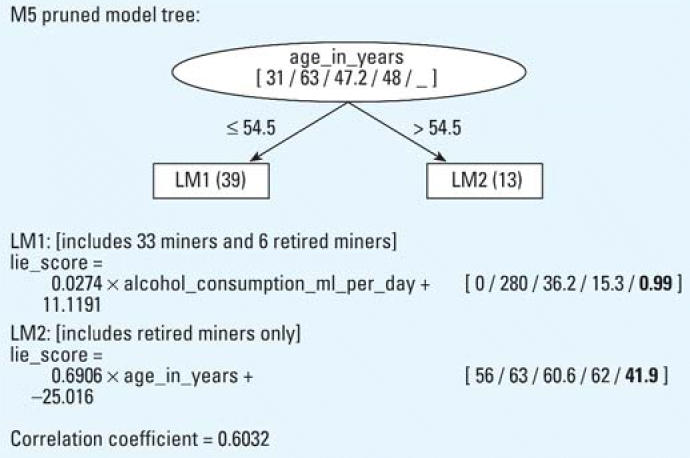
Model tree (with two linear regression models, LM1 and LM2) constructed by M5′, describing the lie score and its correlation coefficient. The numbers in brackets correspond to the minimal, maximal, average, median values, and (in bold if present) the relative importance factor of each numerical attribute. Relative importance factor is the product of an average value and a coefficient in the model.

**Figure 2:**
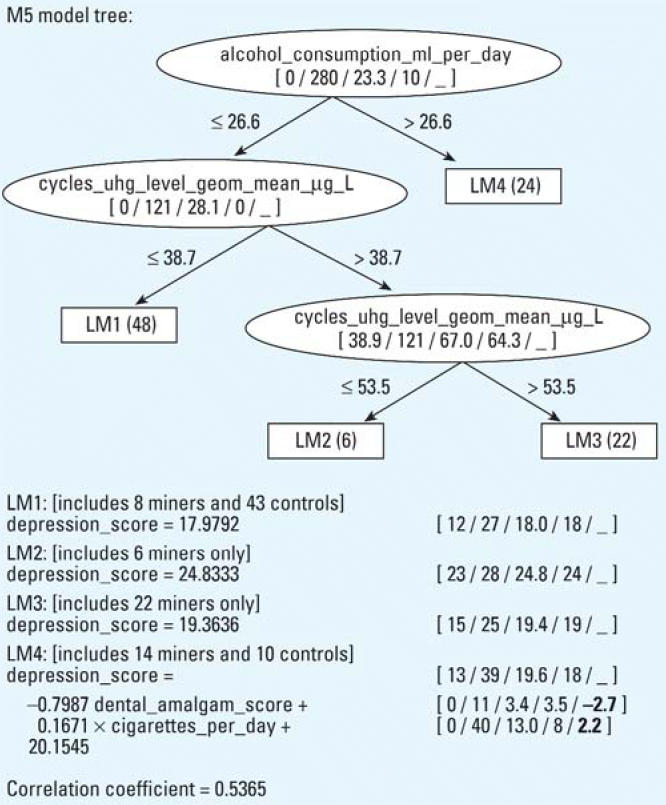
Model tree (with four linear regression models, LM1 to LM4) constructed by M5′, describing the depression score and its correlation coefficient. The number of subjects in each leaf is given in parentheses. The numbers in brackets correspond to the minimal, maximal, average, median values, and (in bold if present) the relative importance factor of each numerical attribute. Relative importance factor is the product of an average value and a coefficient in the model.

**Figure 3:**
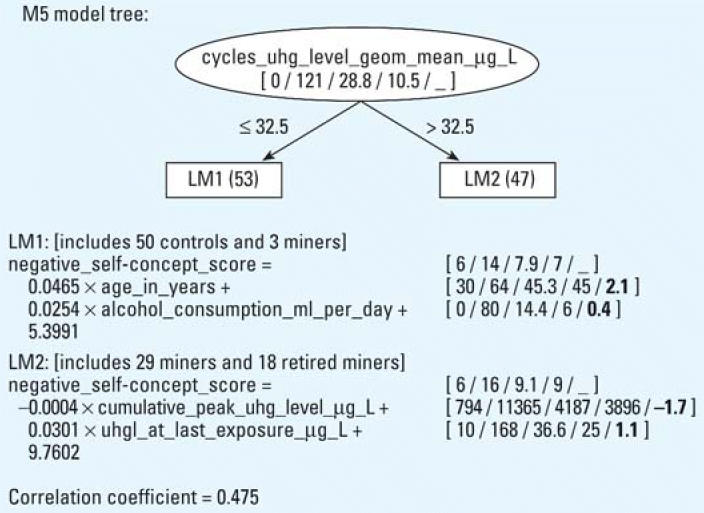
Model tree (with two linear regression models, LM1 and LM2) constructed by M5′, describing the negative self-concept score and its correlation coefficient. The number of subjects in each leaf is given in parentheses. The numbers in brackets correspond to the minimal, maximal, average, median values, and (in bold if present) the relative importance factor of each numerical attribute. The relative importance factor is the product of an average value and a coefficient in the model.

The model trees built for lie, depression, and negative self-concept scores (only target variables with group differences of *p* < 0.01 have been considered) are presented in Figures 1, 2, and 3, respectively.

The model tree predicting the lie score is constructed only on ex-mercury miners. The model tree presented in Figure 1 contains two leaves (linear regression models LM1 and LM2). Alcohol consumption tended to slightly increase the lie score in the subgroup of ex-mercury miners younger than 54.4 years. In the subgroup of older ex-mercury miners, age prominently increased the lie score. No association with external or biological indices of previous exposure is presented in the constructed model tree.

The model tree predicting the depression score, as presented in Figure 2, is constructed on the group of miners and controls. The model tree contains four leaves (linear regression models LM1–LM4), of which three contain constant predictions and one contains a linear model. It is evident from the LM1 model, which was based on a larger number of subjects (39 controls and 9 ex-mercury miners) that low alcohol consumption (< 26.6 mL/day), at lower levels of past ex-miner occupational exposure (mean cycle U-Hg < 38.7 μg/L), did not increase the depression score. Model LM2 relates to the increased depression score in six ex-mercury miners at an intermediate level of exposure (mean cycle U-Hg from > 38.7 to < 53.5 μg/L) and low alcohol consumption (< 26.6 mL/day). The depression score in model LM3, which represents 22 ex-miners with higher past occupational exposure (mean cycle U-Hg > 53.5 μg/L), is not consistent with the expectations observed in model LM2 at an intermediate cycle U-Hg level. The higher consumption of alcohol per se associated with number of cigarettes smoked per day in the model LM4 (> 26.6 mL/day) tends to increase the depression score in 14 ex-mercury miners and 10 controls. If the dental amalgam score is interpreted as the number of repaired teeth improving external appearance (and not as an additional source of occupational exposure), this could be associated with the partial reduction of the depression score among persons in the LM4 model.

In the group with higher alcohol consumption (> 26.6 mL/day) in the LM4 model, the mean GGT concentration in serum (0.99 ± 0.75 μkatal/L) was slightly increased (95th percentile value of the normal male population is 0.92 μkatal/L) and significantly higher (*p* < 0.05) than in the subgroups with lower alcohol consumption (< 26.6 mL/day) in the LM1 (0.45 ± 0.29 μkatal/L) and LM3 (0.45 ± 0.47 μkatal/L) models but statistically insignificantly lower in comparison with the subgroup in the LM2 model (1.44 ± 2.7 μkatal/L). On the basis of mean GGT values in serum, we have assumed that alcohol consumption in these subgroups of ex-miners was also increased during occupational exposure in the past (Joachim 1995).

The model tree predicting the negative self-concept score presented in Figure 3 is constructed on the group of miners and controls. The model tree contains two leaves with one linear model each (LM1 and LM2). Model LM1 represents 50 controls and 3 ex-mercury miners with a relatively low negative self-concept score. Age and alcohol consumption partly increased their negative self-concept score. LM2, which represents ex-mercury miners (*n* = 47, only miners with last exposure U-Hg > 10 μg/L), associates the negative self-concept score with the mean cycle U-Hg level (> 32.5 μg/L) and the U-Hg level at last exposure. The cumulative U-Hg peak level did not increase the observed score.

## Discussion

The present exposure to Hg^0^ was low in both groups and at a level in the general population that is not associated with particular, actual sources of Hg (Minoia et al. 1990; Nordberg et al. 1992; Schaller et al. 1983; Yamamura 1990). The geometric means of cycle U-Hg levels (range, 20–120 μg/L) represent reliable averages of the internal doses of Hg^0^ received by ex-miners during previous occupational exposure. This range of U-Hg levels can be found in many studies (WHO 2003) and, generally speaking, also in our long-term observations (Kobal 1975, 1991; Kobal et al. 1999) associated with neurotoxic effects. Considering the above-mentioned level of Hg^0^ doses received and the results of quoted postmortem studies of ex-mercury miners (Byrne et al. 1995; Falnoga et al. 2000; Kosta et al. 1975), it may be presumed that the accumulation and retention of Hg in the central nervous systems of observed miners during occupational exposure was moderate but characterized by considerable interindividual variability.

The results obtained from the EPQ showed significant differences at the group level between ex-mercury miners and controls. The lower EPQ score of extraversion found in ex-mercury miners suggests that miners are less outgoing and more introverted than the control group. The influence of age on extraversion in males has been reported in some studies (Eysenck and Eysenck 1975; Lojk 1981) but not in ex-mercury miners as opposed to controls (*r* = –0.39, *p* = 0.001). It is evident from the regression tree that the lower score on the lie scale (EPQ) of ex-mercury miners is not associated with indices of Hg^0^ exposure but increases slightly with alcohol consumption and prominently with age in the subgroup of retired miners. A moderate correlation between age and lie score was also observed on the group level (*r* = 0.47, *p* = 0.000). The lower lie score in the subgroup of younger ex-mercury miners (< 54.5 years) suggests that sincerity could be an important personality trait enabling miners to preserve their collaborative and team-working spirit in the mine.

The results obtained from the ESQ also showed significant differences at the group level between miners and the control group. Ex-mercury miners tend to be more rigid in expressing their emotions but are significantly more depressive and are likely to have a more negative self-concept than the members of the control group (*p* < 0.01). The relative indifference (emotional rigidity) expressed by the indifference score seems to be a common characteristic of ex-mercury miners, which correlated well with the depression score of miners (*r* = 0.607, *p* = 0.000). The indifference established in miners in the period after exposure is in genuine contradiction to the known emotional lability that is typical for the state of increased absorption and chronic occupational intoxication with Hg^0^ (Hribernik 1950; Kobal 1975; WHO 2003).

It is evident from the regression tree in Figure 2 that permanent, increased alcohol consumption per se (> 26 mL/day) increases depression in the subgroup of ex-mercury miners and controls, which is also reported in other studies (Leibenluft et al. 1993; Schuckit 1986). Individual susceptibility due to the interaction of Hg^0^ and alcohol could be a possible explanation for the higher depression score observed in 6 ex-miners (with slightly increased GGT) at an intermediate mean cycle U-Hg level (> 38.7 < 53.5 μkatal/L) compared with 22 ex-mercury miners with a higher mean cycle of U-Hg (> 53.5 μkatal/L). Theoretically, such interactions also cannot be completely excluded among ex-miners from the subgroup with higher alcohol consumption. It is known that after inhalation, Hg^0^ enters the bloodstream, where it undergoes oxidation in red blood cells by the hydrogen peroxide catalase pathway (Halbach and Clarkson 1978). The oxidation of Hg^0^ can be inhibited by ethanol (Nielsen-Kudsk 1973), which could increase the accumulation of Hg in the brain (Magos et al. 1989). The differences in enzyme function could be a likely basis for the different response to Hg^0^ in the observed subgroup of ex-miners.

It is evident from the regression tree in Figure 3 that the root node of the tree separated the whole population in the miner and control groups. The internal doses received during past occupational exposure, expressed by a geometric mean of cycle U-Hg level above 32.5 μg/L, seems to be a basic independent variable that separated the two groups and selected 47 ex-mercury miners with a higher negative self-concept. As evident from the constructed model, the cumulative U-Hg peak level is obviously not the most suitable indicator of the total mean internal dose received during past exposure. As mathematic values, the mean cycle U-Hg level and the cumulative U-Hg peak level are in contrast. The first indicator represents the mean of all U-Hg values in which lower values are dominant, and the other indicator represents only the highest values of all exposure cycles.

However, the correlation between depression and negative self-concept (*r* = 0.757, *p* = 0.000) clearly shows that negative self-concept is accompanied with increased depression in subgroups of miners presented in the model tree predicting the depression score. It could be suggested that further investigations including more refined measures of self-concept and self-esteem, and measures of affect (positive and negative) as possible moderators, buffers, or mediators of personality Hg exposure relationship should be done.

Scopoli (1771) precisely described the symptoms and signs of occupational poisoning with Hg^0^ and specifically mentions the “unusually sad mental state of these workers.” Alternations of emotional state, mood, and some unspecific symptoms were most frequently observed at U-Hg levels ranging from 30 to 100 μg/L (Piikivi and Hänninen 1989; Piikivi et al. 1984; Roels et al. 1985; WHO 1991), whereas in some studies these were also observed at lower levels of occupational exposure, that is, at U-Hg mean levels ranging from 30 to 40 μg/L (Echeverria et al. 1995; Soleo et al. 1990). In the study by Soleo et al. (1990) and Echeverria et al. (1995), the personalities of exposed workers were considerably changed at lower levels of occupational exposure, whereas certain mood measures were associated with Hg exposure. In patients not occupationally exposed to Hg^0^ who attributed their illness to Hg from amalgam fillings, a subtle preclinical effect on mood (Echeverria et al. 1998), depression, less extraversion, and more emotional lability were detected (Grandjean et al. 1997). Only a few investigations using the measurements of neuropsychologic effects to study workers previously exposed to Hg^0^ are available to our knowledge (Kishi et al. 1994; Letz et al. 2000; Mathiesen et al. 1999). Mood scales (tension, depression, anger, fatigue, and confusion) were applied only in the study of Letz et al. (2000), but no residual mood changes with depression have been observed.

Any disturbance of the balance between serotonin and melatonin can, in the opinion of certain researchers (van Heeringen 2001), influence the occurrence of depression. Some studies have reported a decreased nocturnal melatonin concentration in the blood of depressed patients (Beck-Friis et al. 1985; McIntyre et al. 1986; Wetterberg 1985). In our ex-mercury miners, however, we detected precisely the opposite, that is, an increased concentration of melatonin in morning blood samples (Kobal et al. 2004). Theoretically, consideration should also be given to the potential impacts of Hg on the metabolism of neurotransmitters (Mottet et al. 1997) and the impacts of increased accumulation of Hg in the pineal gland itself (Falnoga et al. 2000; Kosta et al. 1975), which might also influence the synthesis of melatonin and, indirectly, the balance of serotonin and melatonin (Kasper et al. 1996). In evaluating the potential synergistic neurotoxic impacts of alcohol and Hg, which, in the opinion of certain authors (Halliwell and Gutteridge 1989; Lund et al. 1993; Mottet et al. 1997), are connected with the increased production of free radicals, we must nevertheless consider its interaction with antioxidative enzyme catalase in erythrocytes (Halbach and Clarkson 1978).

This depressive mood itself, especially in association with negative self-concept, could consequently increase the risk of suicide among miners of the Idrija Mercury Mine. Other studies (Kobal Grum 2003) also indicate that a low or negative self-concept could be a significant factor for suicidal behavior. Researchers exploring this variable have maintained that poor self-concept can lead to self-loathing and to consideration of suicide (Harter and Marold 1994). However, the increased mortality due to suicide among miners of the Idrija Mercury Mine in the last 45 years (period of follow-up, 1950–1995; number of miners, 1,589; number of observed suicides, 40; standardized mortality ratio, 123; 95% confidence interval, 88–168; unpublished data) cannot completely confirm the relation between occupational exposure to Hg^0^ in interaction with permanent alcohol consumption and depression with associated negative self-concept as one of the potential causes of suicidal behavior. This is primarily because the results of the epidemiologic study on the mortality of miners in four mercury mines (Boffetta et al. 1998) are not consistent, probably due to errors in the classification of the cause of death in some countries, or due to variations in psychosocial or genetic risk factors (Marušič and Farmer s 2001). Despite the inconsistent results of the above-mentioned epidemiologic study, our assumption is also supported by a rough comparison of the share (%) of ex-miners in the subgroup with increased depression (considering only the six miners with moderate Hg^0^ exposure and moderate alcohol consumption), which is slightly above 11%, and the share (%) of suicides resulting in death among the miners of the Idrija Mercury Mine in the past 45 years, which has attained 2.5%.

## Conclusions

The results obtained in the present study have shown some significant differences, on the group level, in the personality traits of ex-mercury miners compared with controls. Ex-mercury miners are less extraverted, more sincere, more rigid in expressing their emotions, more depressive, and have a more negative self-concept. The lack of correlation between the established target variables of personality traits of ex-mercury miners and the indicators of past occupational exposure on the group level, and the small number of persons in certain subgroups constructed in model trees are the main obstacles preventing the more reliable interpretation of results obtained. Despite these limitations, the above-mentioned theoretical outline and our results suggest that permanent higher alcohol consumption per se, at low levels of Hg^0^ exposure, and the mutual interaction of long-term increased exposure to Hg^0^ with long-term moderate alcohol consumption have presumably had a decisive influence on the development of depression associated with negative self-concept in the subgroup of ex-mercury miners observed. This could, together with other psychosocial factors, be one of the potential reasons for the higher rate of suicide among miners of the Idrija Mercury Mine observed in the last 45 years.

The study thus provides further support to the efforts of other studies aimed at reducing the occupational exposure levels of Hg^0^ to the lowest observed adverse effect level (United Nations Environment Programme 2002; WHO 2003) capable of preventing not only the actual effects on the central nervous system but also the late effects of Hg^0^ exposure on the potential development of depression and negative self-concept, which can significantly decrease resistance toward psychosocial stress factors. The results of the present study suggest that alcohol consumption, although moderate, is not always beneficial and could be avoided during actual occupational exposure to Hg^0^.

## Figures and Tables

**Table 1:** Reliability coefficients of ESQ.

Behavior characteristics	Cronbach α	Guttman split-half
Depression	0.85	0.83
Contentment	0.86	0.87
Aggression	0.68	0.65
Indifference	0.67	0.67
Positive self-concept	0.71	0.65
Negative self-concept	0.71	0.73
Total	0.89	0.84

**Table 2:** Characteristics of observed groups.

	Miners (*n* = 53)	Controls (*n* = 53)	
Characteristics	Mean ± SD	Mean ± SD	*p*-Value
Age	47.32 ± 9.90	44.64 ± 8.54	0.072
BMI (kg/m^2^)	27.8 ± 4.1	27.4 ± 4.1	0.536
Dental amalgam score	12.8 ± 12.4	12.5 ± 10.9	0.912
Fish meals/week	0.52 ± 0.96	0.59 ± 0.88	0.649
Cigarettes/day^a^	21.6 ± 7.3	20.5 ± 9.5	0.693
Alcohol (mL/day)^b^	35.2 ± 40.2	22.4 ± 18.6	0.099
B-THg (μg/L)	2.5 ± 1.5	2.5 ± 1.2	0.158
U-Hg (μg/g creatinine)	2.1 ± 1.4	1.4 ± 1.1	0.003

a Percentage of smokers: miners, 59%; controls, 41%.

b Percentage of alcohol consumers > 20 mL/day: miners, 28%; controls, 19% (*p* > 0.05). Data from Kobal et al. (2004).

**Table 3:** External and biological indices of previous occupational Hg^0^ exposure in miners (*n* = 53).

Indices	Geometric mean ± SD	Range
Years of exposure	14.6 ± 5.5	7–31
Cycles of exposure	41 ± 21	13–119
ATW air Hg^0^ concentration (mg/m^3^)	0.29 ± 0.08	0.14–0.45
Cycle U-Hg level (μg/L)	53.1 ± 20.5	20–120
Cycle peak U-Hg level (μg/L)	77.2 ± 23.0	40–134
Cumulative U-Hg level (μg/L)	6,584 ± 4,444	1,286–21,390
Cumulative peak U-Hg level (μg/L)	3,900 ± 2,196	794–11,365
Last exposure U-Hg level (μg/L)	26 ± 29	8–135

**Table 4:** Average scores on the EPQ of observed groups.

	Miners (*n* = 53)	Controls (*n* = 53)	
EPQ	Mean ± SD	Mean ± SD	*p*-Value
Psychoticism	3.80 ± 2.37	3.74 ± 2.06	0.905
Extraversion	12.09 ± 3.81	13.93 ± 3.15	0.017
Neuroticism	8.14 ± 4.27	7.55 ± 4.21	0.522
Lie	12.45 ± 4.22	15.05 ± 3.54	0.003

**Table 5:** Average scores on the ESQ of observed groups.

	Miners (*n* = 53)	Controls (*n* = 53)	
ESQ	Mean ± SD	Mean ± SD	*p*-Value
Depression	20.33 ± 5.07	17.73 ± 3.61	0.009
Contentment	30.52 ± 4.97	31.05 ± 6.06	0.667
Aggressions	17.17 ± 4.20	15.95 ± 2.71	0.122
Indifference	9.74 ± 2.73	8.51 ± 2.11	0.025
Positive self-concept	15.52 ± 3.16	16.51 ± 2.91	0.143
Negative self-concept	8.98 ± 2.51	7.68 ± 1.71	0.008
